# Hematological, Biochemical, Histopathological and ^1^H-NMR Metabolomics Application in Acute Toxicity Evaluation of *Clinacanthus nutans* Water Leaf Extract

**DOI:** 10.3390/molecules23092172

**Published:** 2018-08-29

**Authors:** Leng Wei Khoo, Audrey Siew Foong Kow, M. Maulidiani, Ming Tatt Lee, Chin Ping Tan, Khozirah Shaari, Chau Ling Tham, Faridah Abas

**Affiliations:** 1 Department of Food Science, Faculty of Food Science and Technology, Universiti Putra Malaysia, 43400 Serdang, Selangor, Malaysia; lengweikhoo@gmail.com; 2 Department Biomedical Science, Faculty of Medicine and Health Sciences, Universiti Putra Malaysia, 43400 Serdang, Selangor, Malaysia; audrey_k_84@yahoo.com (A.S.F.K.); chauling@upm.edu.my (C.L.T.); 3 Laboratory of Natural Products, Institute of Bioscience, Universiti Putra Malaysia, 43400 Serdang, Selangor, Malaysia; dieni_maulydia@yahoo.com (M.M.); khozirah@upm.edu.my (K.S.); 4 Faculty of Pharmaceutical Sciences, UCSI University Kuala Lumpur Campus, Jalan Menara Gading, UCSI Heights (Taman Connaught), Cheras, 56000 Kuala Lumpur, Malaysia; leemt@ucsiuniversity.edu.my; 5 Department of Food Technology, Faculty of Food Science and Technology, Universiti Putra Malaysia, 43400 Serdang, Selangor, Malaysia; tancp@upm.edu.my

**Keywords:** *Clinacanthus nutans*, medicinal plant, biochemical test, histopathology, urinary metabolomics, toxicity, ^1^H-NMR metabolomics, metabolic pathway

## Abstract

The present study aims for the first time to provide the in vivo acute toxicological profile of the highest dose of *Clinacanthus nutans* (Burm. f.) Lindau water leaf extract according to the Organization for economic co-operation and development (OECD) 423 guidelines through conventional toxicity and advanced proton nuclear magnetic resonance (^1^H-NMR) serum and urinary metabolomics evaluation methods. A single dose of 5000 mg/kg bw of *C. nutans* water extract was administered to Sprague Dawley rats, and they were observed for 14 days. Conventional toxicity evaluation methods (physical observation, body and organ weight, food and water consumption, hematology, biochemical testing and histopathological analysis) suggested no abnormal toxicity signs. Serum ^1^H-NMR metabolome revealed no significant metabolic difference between untreated and treated groups. Urinary ^1^H-NMR analysis, on the other hand, revealed alteration in carbohydrate metabolism, energy metabolism and amino acid metabolism in extract-treated rats after 2 h of extract administration, but the metabolic expression collected after 24 h and at Day 5, Day 10 and Day 15 indicated that the extract-treated rats did not accumulate any toxicity biomarkers. Importantly, the outcomes further suggest that single oral administration of up to 5000 mg/kg bw of *C. nutans* water leaf extract is safe for consumption.

## 1. Introduction

*Clinacanthus nutans* (Burm. f) Lindau, commonly known in Malaysia as Sabah snake grass or ‘*belalai gajah*’ is a well-known traditional herb and vegetable that grows wild or is cultivated mostly in Southeast Asia countries. This plant has a long history of use for treating snake bites, herpes infection, skin infection, cancer, burns and scalds, dysentery, and diabetes in Thailand, Indonesia and Malaysia [1]. Since 1987, *C. nutans* has been one of the appointed medicinal herbs used in hospitals under the program of primary healthcare by the government of Thailand [1]. In recent years, *C. nutans* has also been classified as the initial focus under Entry Point Project (EPP) by the government of Malaysia [2]. The practice of utilizing herbal plants as medicines in the form of tinctures, elixirs, poultices, powders and other herbal formulations for chronic disease management or health boosting purposes has occurred since the beginning of civilization and becomes increasingly common in our society [3]. However, the incorrect use and the lack of scientifically backed information, such as the authentication of the plant material and its active principles, prescription method, efficacy, bioavailability and the toxicant compositions, appropriate concentrations and mechanisms of medicinal plants may lead to detrimental effects on our bodies to the point of death. Hence, feasible and efficient toxicity testing is important in evaluating the potential toxic effect of any substances before they are deemed safe to be consumed by humans [4]. 

Toxicity tests are divided into acute, subacute and chronic tests. Acute toxicity testing is a short-term toxicity evaluation using a single dose of the test substance, and it is also known as the LD_50_ (dose that kills 50% of animals) [4]. A lot of information can be extracted from this testing, including identification of the target organ of toxicity, safety and monitoring guidelines for workers involved in developing and testing of test substances, dose selection in prolonged toxicity studies, data on adverse effects on humans, animal health and environment, guidance for other testing programs, and the classification, labelling and transportation of chemical agents [4]. To date, a number of acute toxicity profiles of *C. nutans* extracted by different polar solvents have been established. These include profiles of butanol [5], ethanol [6], methanolic [7,8], and water [9] extracts. Thus far, all the previous toxicity studies have reported that *C. nutans* is safe for consumption.

Nevertheless, to the best of our knowledge, there is a lack of toxicological studies that have assessed the biological endogenous metabolites variations in rats after *C. nutans* extract administration. To assure the safety fact of natural products, a more comprehensive understanding of toxicity is urgently needed. Metabolomics is a high-throughput omics-technique that is well-suited for this purpose. Metabolomics, which is a holistic approach, is in agreement with natural herbal medicine theory [3]. Metabolomics attempts to systematically profile, quantify and integrate a large number of low-molecular weight endogenous metabolites that can illustrate the fundamental metabolism status of a body. Any perturbation that affects a biological system can be reflected by the metabolome and revealed the metabolic characteristics of biological samples [3,10,11]. This modern technology possesses an important advantage over conventional methods because the metabolome represents the physiological or pathological status of organisms, and thus, metabolomics-based toxicology is of significance to detect abnormal changes in endogenous metabolites and identify toxicological biomarkers that are present before toxins have caused physiological or pathological damages [3]. Hence, in recent years, there has been emerging interests in extending the application of metabolomics to the field of toxicology [10]. 

Therefore, the present study aims to evaluate and confirm the acute toxicity profile of 5000 mg/kg of *C. nutans* water leaf extract after single-dose oral administration. Both conventional physical observation and hematological, biochemical and histopathological analytical methods together with 500 MHz high-resolution proton nuclear magnetic resonance (^1^H-NMR) metabolomics approach were applied in this study to examine the possible physical or endogenous metabolites changes in organ, serum or urine samples before and after oral administration of *C. nutans* water leaf extract. 

## 2. Results and Discussion

### 2.1. Physical and Behavioral Changes Related to Acute Oral Toxicity 

In the present study, the toxicity of *C. nutans* water leaf extract was assessed because the water extract is the most commonly used form. Meanwhile, acute oral toxicity was chosen to identify a safe dose for the following study in which *C. nutans* water extract will be given orally once throughout the period of study. Therefore, subacute, subchronic and chronic toxicity studies do not fall within the scope of our study. 

**Figure 1:**
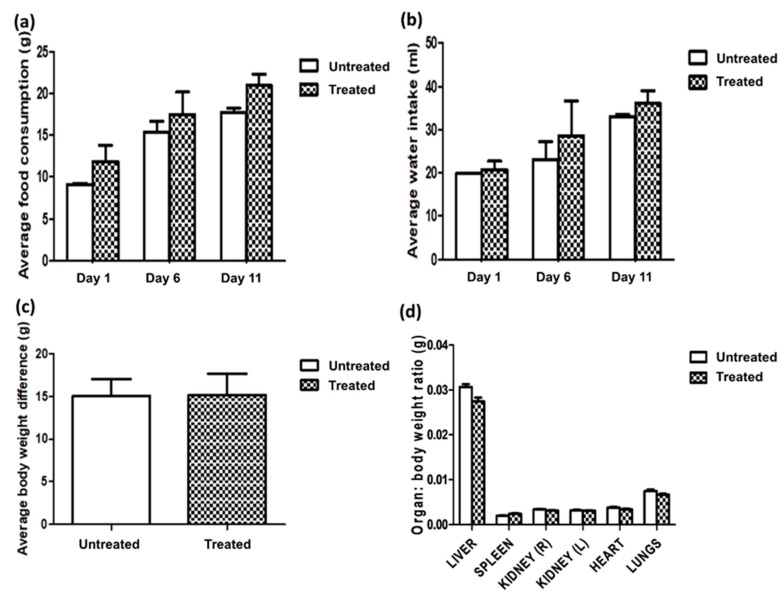
(**a**) Average food consumption, (**b**) average water intakes, (**c**) average body weight and (**d**) relative organ weight to body weight ratio of liver, spleen, kidneys, heart and lungs of untreated rats and rats given 5000 mg/kg of *C. nutans* water leaf extract.

Cage-side observation for signs of toxicity was performed with specific attention given in the first 6 h after administration of *C. nutans* water extract (5000 mg/kg) and then once daily for 14 days. No physical changes were observed in the untreated or treated animals. There were no signs of inactivity, isolation or scratching. All animals were walking normally, i.e., no limping was observed; animals ate and drank as usual; and no diarrhea was observed. Both food intake and water consumption were recorded every five days after administration of the test substance, and the body weights of all animals were recorded on Day 0 and Day 15. There was no significant difference in the food intake, water consumption and body weight between the two groups of animals (Figure 1). It was also noted that there was an increase in their consumption and body weight of all animals throughout the 14-day period. This showed that the extract did not affect the appetite of the animals in general.

Overall, all the animals survived the 14-day period of the study, i.e., no signs of toxicity or mortalities were recorded. This is also supported by the study of Farsi et al. [9] in which no morbidity, mortality or apparent signs of toxicity were observed. Hence *C. nutans* water extract was considered safe at an acute dose of 5000 mg/kg in accordance with the OECD guideline. 

### 2.2. Hematology and Biochemical Analysis

Hematology analysis was performed to evaluate the effect of *C. nutans* water extract on the full blood profile and to evaluate its effect on white blood cells components, which are the first line of our cellular defense against foreign particles [12]. As observed in Table 1, there were no significant differences in most of the hematology parameters analyzed except the packed cells volume (PCV) and mean corpuscular hemoglobin concentrations (MCHC). A low PCV usually indicates blood loss due to cell destruction or failure in bone marrow production, while high MCHC indicates the presence of spherocytes in the blood, which could be a sign of anemia [12]. However, both values were still within the normal range of 35.2–49.8% (0.352–0.498 L/L) for PCV and 322.0–367.0 g/L for MCHC [13]. In addition, hemolysis could have contributed to a false increase in MCHC and decrease in PCV [12]. Hence, it is safe to say that *C. nutans* water extract did not affect the blood profile of the animals, further ensuring the safety of this extract even at a high dose of 5000 mg/kg.

Biochemical analysis can provide data that indicate possible damaging effects of *C. nutans* water extract on liver and kidney functions. The liver is the primary drug metabolizing organ and plays an important role in the elimination of drugs [14]. Liver enzymes such as aspartate aminotransferase (AST), alanine aminotransferase (ALT), alkaline phosphatase (ALP) and gamma-glutamyl transferase (GGT) are useful markers of hepatocellular damage; any increase in the levels of these markers indicates liver disease [14]. Under normal conditions, these enzymes are stored in the cytosol, and they will be released into the blood if the liver is injured by toxic substances or other causes [15]. Total bilirubin, on the other hand, reflects liver problems such as jaundices, hepatitis and gallstones. An elevated level of bilirubin will cause an intense yellow color to be observed in the urine [15]. Hepatotoxicity is also linked to the clinical manifestations of drug allergy such as fever, rash and eosinophilia [14]. As shown in Table 2, there was no significant difference in AST, ALT, ALP, and total bilirubin between the untreated and treated animal groups. In addition, there were no signs of liver enlargement or increment in the weights of the livers of the animals. 

Kidneys are also involved in the metabolism, detoxification, storage and excretion of foreign materials [16]. Parameters such as urea and creatinine levels are analyzed to evaluate the toxic effect of a substance. Any elevations in the levels of these parameters are indicative of nephrotoxicity. In our study, there was no significant difference between the two groups of animals in either of the parameters analyzed (Table 2). Farsi et al. [9] also found that *C. nutans* water extract at 5000 mg/kg did not affect the levels of urea and creatinine. There was no enlargement in the kidneys from any of the animals, and there was no significant difference in the relative organ weight recorded. Hence, at 5000 mg/kg, *C. nutans* water extract was found to be nontoxic to the liver or kidneys of the animals. 

### 2.3. Histopathological Analysis

**Figure 2:**
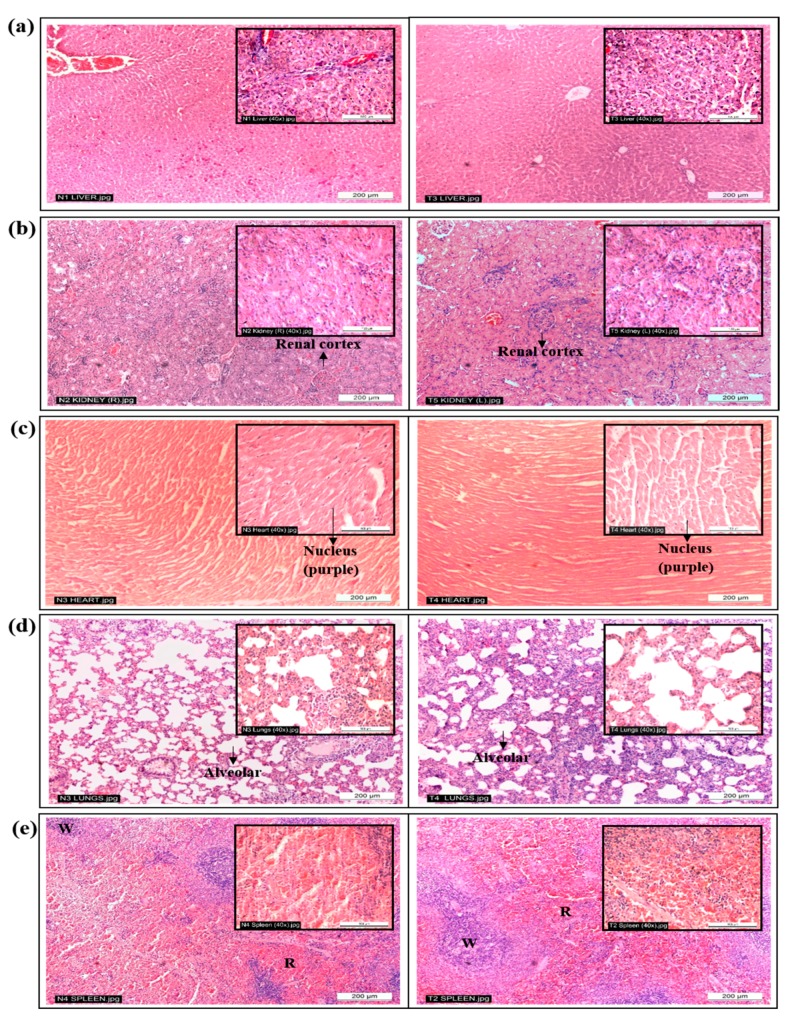
Representative photographs of histological sections of (**a**) liver, (**b**) kidney, (**c**) heart, (**d**) lung and (**e**) spleen tissues of rats given distilled water (**left**) and 5000 mg/kg of *Clinacanthus nutans* water leaf extract (**right**) showing normal architectures (H & E ×100 magnification; Inset: ×400 magnification). White pulp (W); Red pulp (R).

Histopathological analysis, which involved both macroscopic and microscopic examinations of the lungs, heart, spleen, kidneys and liver further supported the biochemical analysis done. Microscopically, no abnormalities were observed in the branches of hepatocytes cords in the livers (Figure 2a). In a normal liver, the hepatocytes are able to anastomose freely, forming a labyrinthine and sponge-like structure, while the liver sinusoids are composed of irregularly dilated vessels with a discontinuous fenestrated layer of endothelial cells [17]. In addition, there were no structural differences observed in the kidneys’ renal cortex (Figure 2b). The renal tubules are considered normal if they are coated by a single layer of cuboid cells densely populated with eosinophils and the tubules are optically empty [18]. 

Microscopic observation of the hearts from both groups of animals showed normal myocardial fibre appearances, as depicted in Figure 2c. Structurally, a normal heart will have well-connected intercalated discs, and the filaments are normally arranged with no swelling [19]. Damaged muscle fibres in the heart will usually fluoresce under hematoxylin and eosin staining [19], and this was not reflected in any of the animals. No signs of inflammation or increased interstitial space were observed in the heart tissues of the treated animals. On examination, the lungs of the treated animals (Figure 2d) appeared normal with thin inter-alveolar septa, normal clear alveoli and alveolar sacs, and there was no presence of inflammatory cells, such as neutrophils, or thickening of the alveolar septa [20]. Lastly, there were no differences between the spleens of the animals (Figure 2e). Spleen tissues that are damaged due to toxic substances will be presented with activated germinal centres of the white pulp, megakaryocytosis and eosinophil-filled red pulp [21]. However, these were not observed in the histopathological analysis of the spleens of treated animals; no disruption in the microstructure nor the presence of inflammatory cells, edema or necrosis were noted, and all the white and red pulps were enclosed by a capsule of dense connective tissue [21]. Likewise, there was no significant difference in the relative organ weight (Figure 1) of all the organs between the untreated and treated animal groups, which further supported the microscopic observations of the individual organs, whereby all showed normal architecture. Moreover, no leukocytes infiltration was noted. 

Hence, *C. nutans* water extract at 5000 mg/kg was not toxic to the heart, lungs, kidneys, liver or spleen of female Sprague Dawley rats. In addition, based on previous acute oral toxicity studies, a single dose of *C. nutans* extract showed no or very low toxicity in the animals. The LD_50_ of *n*-butanol extract was 13.40 ± 1.6 g/kg when the rats were administered via oral gavage and 3.40 ± 0.1 g/kg when administered intraperitoneally [5]. Methanolic extract of *C. nutans* did not showed any signs of toxicity at 2000 mg/kg when administered to Sprague Dawley rats [7] or at 1800 mg/kg when administered to male Swiss albino mice [8]. Furthermore, Chavalittumrong et al. [6] found that mice given 1.3 g/kg of ethanolic *C. nutans* extract orally, subcutaneously or intra-peritoneally did not show any signs of toxicity throughout the 15-day observation period. With all these findings, it can be safely said that *C. nutans* is nontoxic for human consumption even at the highest dosage recommended by OECD.

### 2.4. ^1^H-NMR Serum Metabolomics Evaluation

The results obtained from the conventional physical observation and hematological, biochemical and histopathological analyses suggest that *C. nutans* is safe for single-dose consumption even at the highest recommended dosage of 5000 mg/kg bw in rats. Further analysis of the bio-fluid (serum and urine) samples using ^1^H-NMR spectroscopy was carried out to confirm the results obtained above as well as to study the possible metabolic variation related to toxicity signals. The metabolites detected in serum samples were identified based on the ^1^H-NMR spectra of both untreated and treated rats’ serum collected the day before treatment (Day 0) and at the end point of the experiment (Day 15) (Table S1). In total, 22 primary metabolites plus an unknown signal (δ1.18) were successfully identified. The metabolites were assigned by referring to Chenomx software databases (Version 8.1, Alberta, Canada), electronic human metabolome databases (HMDB) (http://www.hmdb.ca/) [22], and literature data [11,23]. Visual comparison of ^1^H-NMR spectra of the untreated and treated groups did not demonstrate significant differences.

**Figure 3:**
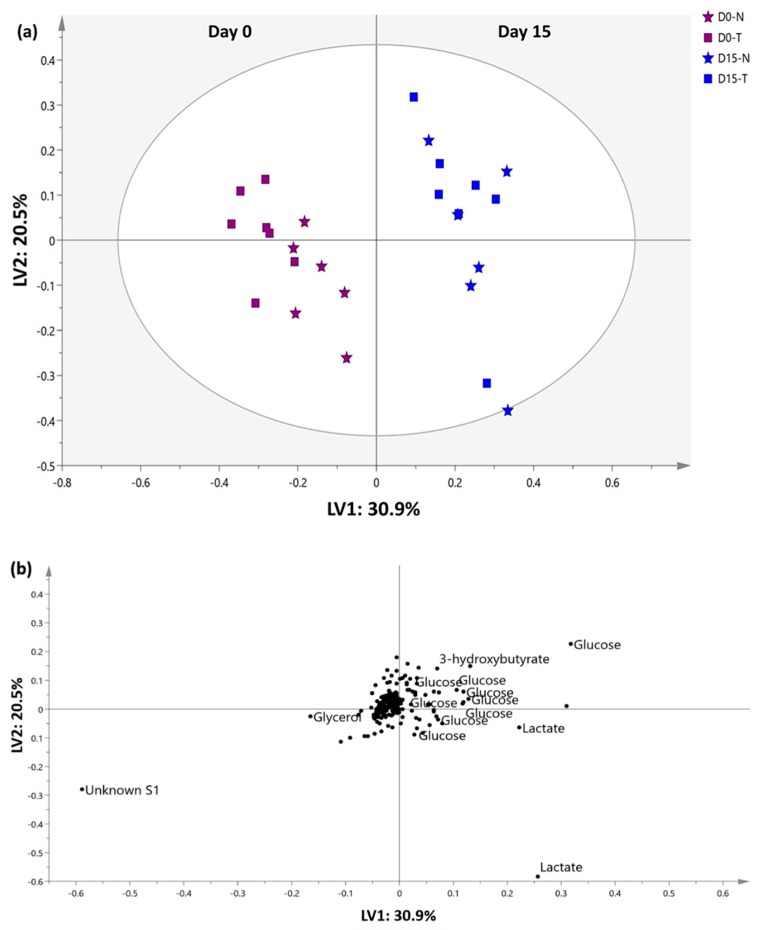
PLS-DA (**a**) score and (**b**) loading plot of serum of untreated and treated rats collected on Day 0 and Day 15. N: untreated rats, T: treated rats.

A multivariate data analysis (MVA) tool was then coupled with ^1^H-NMR data to efficiently establish a relevant summary from the ^1^H-NMR spectra data for serum and to minimize any possible biases. Partial least squares discriminant analysis (PLS-DA), a supervised projection model, was performed as shown in Figure 3 to discriminate the metabolic phenotypes and the metabolite biomarker changes in serum of untreated and treated groups that was collected on Day 0 and Day 15. Model validation, such as cross validation and response permutation testing, was performed and it validated that the generated PLS-DA model exhibited a good performance and predictive ability (R^2^Y = 0.931, Q^2^Y = 0.865, cross validation analysis of variance (CVANOVA) with *p* < 0.05, new estimated R^2^Y intercept value < 0.5 and new permuted Q^2^Y intercept < 0.05) [24].

The first two latent variables of the PLS-DA model cumulatively accounted for 51.4% of the total variance (first latent variable (LV1) with 30.9% and LV2 with 20.5%) and contributed to the most variation. Ideally, serum collected on Day 0 from untreated and treated rats was expected to have high similarity in terms of the endogenous metabolites present. Analysis of serum collected on Day 15, on the other hand, is important to determine whether any possible perturbation occurred in the metabolism status of the rats after extract administration. From the PLS-DA score plot, it can be observed that the treated and untreated groups shared a high percentage of similarity because they were closely clustered even after 14 days of the experiment. Compared with Day 0, serum from untreated and treated rats collected on Day 15 contained more glucose, lactate, and 3-hydroxybutyrate. Glucose, fatty acids, ketone bodies, lactate and pyruvate are among the common substrates used for energy production [25]. It is hypothesised that the rats which were at approximately 12 weeks old on Day 15 had higher growing rate. Higher activity and metabolism rate were experienced, and higher nutrient requirement was observed (as showed in Figure 1) compared to Day 0. As a consequence, elevated 3-hydroxybutyrate, glucose and lactate, the metabolic products of ketogenesis and gluconeogenesis might indicate higher metabolic activity and fasting experienced prior to blood collection [26,27].

Liver biochemical tests assessed the ALT, AST and ALT enzyme levels in the serum as an indication of liver damage due to toxicity, and ^1^H-NMR metabolomics was used to evaluate the toxicity effect of the extract based on the metabolic products released by the liver into the blood. Overall, metabolic products such as pyruvate and l-glutamate (released as a resultant of the enzymatic activity of ALT), oxaloacetate and l-glutamate (from l-aspartate and 2-oxoglutarate catalyzed by AST), alcohol and phosphate (catalyzed by ALP from phosphate monoester), and bilirubin, were absent or not significantly present in extract-treated rats. The ^1^H-NMR metabolomics results were in agreement with the results of the conventional acute toxicity diagnosis methods. Both the untreated and treated groups were found to possess similar serum metabolomes profiles after 14 days of observation. Thus, collected urine was further analysed to justify the statement.

### 2.5. ^1^H-NMR Urinary Metabolomics Analysis with Suggested Metabolic Pathways

Examination of the serum metabolome did not reveal any significant xenobiotics. Urine is one of the main excretion routes for toxicant metabolic products to be eliminated from the body. More metabolites are therefore expected to be detected, and any toxicity caused by the extract will be reflected in the urine metabolome profile in response to injury. The metabolites identified in urine from both untreated and treated rats were based on the ^1^H-NMR spectra collected before treatment (Day 0), the treatment day (Day 1), post-treatment days (Days 2, 5, and 10) and the final time point (Day 15) (Table S1). In total, 36 metabolites were identified, including two unknowns (labelled unknown U1 and U2), which were found to be significant in the treated group (Table S1). 

**Figure 4:**
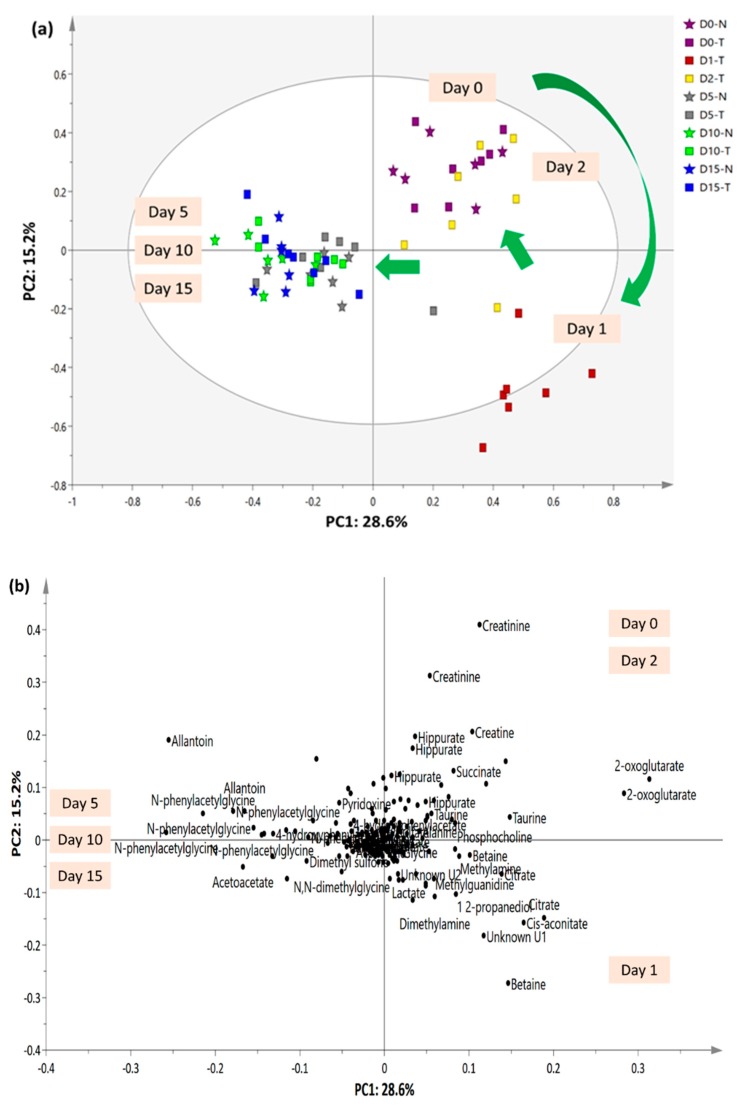
Principal component analysis (PCA) (**a**) score and (**b**) loading plots of urinary profiles trajectory trend of the untreated and treated groups collected on Day 0, 1, 2, 5, 10 and 15.

Overall visual inspection of the ^1^H-NMR spectra suggests that the urinary metabolites collected on Day 1 exhibited more variation compared with those in urine collected on the other days. The MVA tool further examined the discriminating patterns and the changes in urinary metabolites after treatment with the extract. The overall urinary profile trajectory trend of the untreated and treated groups on Days 0, 1, 2, 5, 10 and 15 was successfully illustrated by a principal component analysis (PCA) score plot model, as shown in Figure 4. Urine collected from the treated groups on Days 0, 1 and 2 showed more metabolite differences than urine collected on Days 5, 10 and 15 and thus was separated by PC1. Generally, untreated and treated rats exhibited close clustering on Day 0. After 2 h (Day 1), significant metabolite variation was observed in the urine collected from the extract treated group. Nevertheless, after 24 h of extract administration (Day 2), the urine of treated rats was closely grouped with urine collected on Day 0 from the untreated and treated groups. Further evaluation of the urinary metabolites collected on Days 5, 10 and 15, as described in the negative PC1, suggests that the untreated and extract-treated groups on post-treatment days shared high similarity and they possessed higher allantoin, *N*-phenylacetylglycine, acetoacetate, 4-hydroxyphenylacetate, dimethyl sulfone, *N*,*N*-dimethylglycine, pyridoxine, acetic acid, trigonelline and 3-indoxysulfate. In addition, excessive removal of creatinine and urea was not observed in the urine, which aligned with the results of the kidney function biochemical tests. 

**Figure 5:**
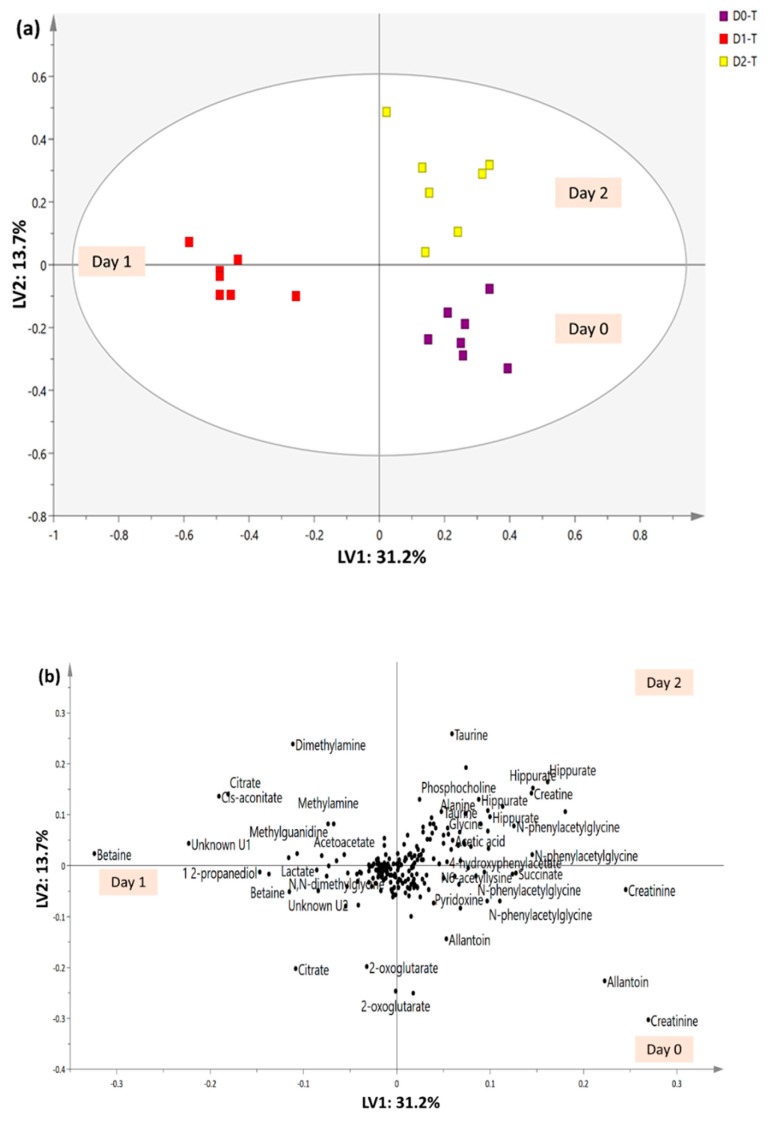
PLS-DA (**a**) score and (**b**) loading plots of urinary profiles trajectory trend of the treated groups collected on Day 0, 1, and 2.

Nevertheless, urine collected from treated rats on Day 1 possessed the most variation. The PLS-DA model was further constructed to classify the metabolic pattern among the urine collected from treated group on Days 0, 1, and 2 and to assure that the differentiating metabolites did not have an acute drastic effect on the rats. The model was validated by cross validation and permutation tests and considered as a good performance model according to Eriksson’s theory. Cross validation showed a parameter R^2^Y value of 0.862, Q^2^Y value of 0.762, and CVANOVA with *p* < 0.05. A permutation test revealed that a new permuted R^2^Y intercept < 0.30 and Q^2^Y intercept < −0.25. Similar to Figure 4, Figure 5 demonstrates that urine samples collected on Day 1 and samples collected on Days 0 and 2 were located in two opposite directions, separated by LV1. The PLS-DA loading plot indicates that betaine, cis-aconitate, citrate, dimethylamine, 1,2-propanediol, methylguanidine, methylamine, lactate, *N*,*N*-dimethylglycine, and acetoacetate, together with unknown U1 and U2, were the main metabolite differentials in treated rats after 2 h of extract administration. Thus, these metabolites were relatively quantified, and the metabolites levels and fold changes are summarized in Table 3.

**Figure 6:**
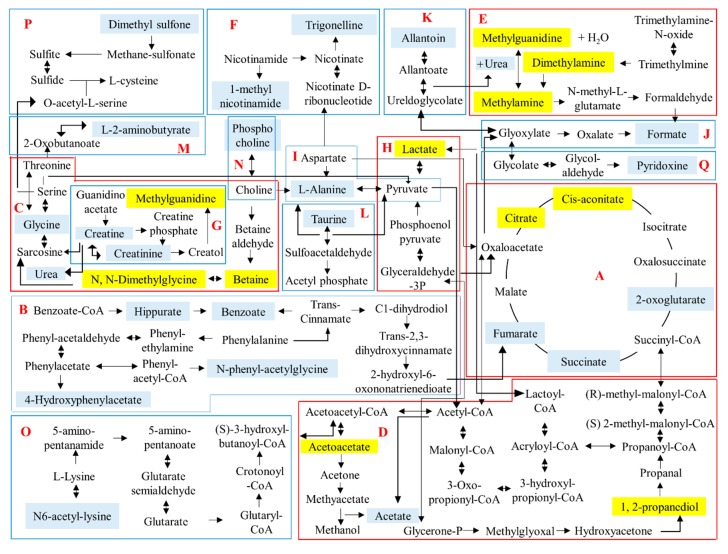
The schematic diagram of the suggested metabolite pathway based on urine collected from both untreated and treated rats. The metabolites highlighted in blue are metabolites successfully identified from the urine of both untreated and treated rats. The metabolites highlighted in yellow are metabolites that showed significantly greater intensities in urine of treated rat collected on Day 1. A: TCA cycle, B: Phenylalanine metabolism, C: Glycine, serine, threonine metabolism, D: Propanoate metabolism, E: Methane metabolism, F: Nicotinate & nicotinamide metabolism, G: Arginine & proline metabolism, H: Glycolysis, I: Alanine, aspartate & glutamate metabolism, J: Glyoxylate & dicarboxylate metabolism, K: Purine metabolism, L: Taurine & hypotaurine metabolism, M: Cysteine & methionine metabolism, N: Glycerophospholipid metabolism, O: Lysine degradation, P: Sulfur metabolism, Q: Vitamin B6 metabolism.

In total, 17 metabolic pathways that are closely related to the identified urinary metabolites (Table S1) were tabulated in the schematic diagram, as depicted in Figure 6 with the aid of the KEGG online database. Out of the 17 metabolic pathways, a total of five pathways including glycolysis, tricarboxylic acid (TCA) cycle, propanoate metabolism, methane metabolism, and glycine, serine and threonine metabolism pathways were significantly altered in the treated group after 2 h of *C. nutans* water extract administration.

Among the influenced metabolic pathways, carbohydrate metabolism that comprised the TCA cycle, propanoate metabolism and glycolysis was the most affected. The TCA cycle is the final metabolic pathway for glucose, fat and amino acid and is normally used to generate energy and provide major nutrients [11]. Citrate is a weak acid that can be obtained via dietary intake and synthesized via the TCA cycle [11]. Accumulation of citrate level reduced citrate synthase activity and disturbed the energy supply of the TCA cycle [11]. On the other hand, *cis*-aconitate is produced through the dehydration of citric acid and is an intermediate in the TCA cycle [28]. Acetoacetate, which is produced in propanoate metabolism, is one of the main ketone bodies (in addition to 3-*β*-hydroxybutyrate and acetone) found in liver cells. It can be used as an alternative energy source when carbohydrates are scarce. Elevated acetoacetate in the body might be caused by fasting or prolonged exercise [29]. 

1,2-Propanediol is observed in propanoate metabolism and is an abundant fermentation product of the plant sugars l-rhamnose and l-fucose [30]. These two sugars are frequently found in the carbohydrate moieties of mucosal glycoconjugates and in cell walls of food from herbal origin. Notably, 1,2-propanediol was mainly detected in the urine collected on Day 1 and Day 2 from treated rats. It was hypothesized that this metabolite is the metabolic product of the plant extract, and its terminal half-life was between 1.4 to 3.3 h in normal functioning livers and kidneys [22]. Approximately half of 1,2-propanediol is metabolized by the liver to form lactate, acetate, and pyruvate while approximately 12–45% is excreted unchanged in urine. Since it originates from an exogenous source, its presence raises toxicity concerns. According to Kelava et al. [31], the toxicity of 1,2-propanediol is low, with an LD_50_ in rats of 13 mL/kg intraperitoneal (*i.p*.) or 6.2 mL/kg intravenous (*i.v*.) injection. In addition, 1,2-propanediol was reported to delay the onset of seizure [31], show an immunosuppressive effect on macrophages, natural killer cells, and neutrophils [32], possess anti-inflammatory effect in carrageenan-induced edema [31,33] and act on monocytes migration in both pleurisy phases [33]. The observed lactate accumulation revealed the disturbance of glycolysis in the treated rats. As an indicator of anaerobic respiration, increased lactate in urine samples might suggest up-regulated glycolysis in rats exposed to *C. nutans* water extract.

The increment of methylguanidine, dimethylamine, and methylamine in the treated rats suggests that methane metabolism, categorized under energy metabolism was disturbed. Methylguanidine, a guanidine compound, can be synthesized from the reaction between methylamine and urea under methylguanidinase activity [34]. More commonly, methylguanidine is derived from creatinine in protein catabolism. Previous results regarding the function of methylguanidine are contradictory. One study suggested that it was a uremic toxin that accumulated in renal failure [35]. Nevertheless, more evidence suggested that it exhibited anti-inflammatory properties by significantly inhibiting inducible nitric oxide synthase activity and tumor necrosis factor-release [36] and attenuating inflammation and tissue damage in a carrageenan-induced acute inflammation and pleurisy rat model [35]. Dimethylamine and methylamine are biogenic amines with a fish-like odor [37]. They are common urinary metabolites. Urinary dimethylamine which normally originates from trimethylamine-*N*-oxide and asymmetric dimethyl-arginine, is an endogenous inhibitor of nitric oxide synthesis. Dzhanashvili [38] reported that the oral LD_50_ of dimethylamine was 700 mg/kg bw rat at base pH, while the LD_50_ was 8100 mg/kg bw rat at pH 8.0. Methylamine, the simplest aliphatic amine, can be obtained from fish, seafoods, and some fruits and vegetables. It is an important endogenous source of dimethylamine and methionine [39]. It has important physiological effects on the central nervous system during renal and hepatic disease and plays a role in the general toxicity caused by oxidative stress [39].

Lastly, betaine, *N*,*N*-dimethylglycine, and methylguanidine, which were perturbed in the urine collected on Day 1 from *C. nutans* treated rats are closely related to glycine, serine, and threonine metabolism. The elevation of these metabolites suggests the enhancement of amino acid metabolism. Betaine, also known as *N*,*N*,*N*-trimethylglycine, is commonly acquired from food or from dietary choline. The overall effect of betaine is unlikely to be harmful and may be beneficial according to previously reported studies. The function of betaine as a methyl functional group donor is important because the donation could facilitate liver function, protect kidneys from damage, promote cellular replication and detoxification and produce *N*,*N*-dimethylglycine [40]. Study also reported that betaine is a potential antioxidant and has an ameliorating effect on the nitric oxide production in activated microglial cells [41]. Conversely, betaine insufficiency will lead to metabolic syndrome, lipid disorders, and diabetes problems [40]. Other significantly altered amino acids in the extract-treated rats include *N*,*N*-dimethylglycine. *N*,*N*-dimethylglycine is a metabolite of betaine and a by-product of homocysteine metabolism. It is a relatively non-toxic substance, and it has been patented as an effective compound for treating arthritis and inflammation [42].

In general, the identified serum and urinary metabolites suggest that the extract did not exhibit hazardous characteristic to the extract treated rats. To date, there are no studies that have isolated any potential toxic materials from *C. nutans*, which further affirms the safety profile of *C. nutans*.

## 3. Materials and Methods

### 3.1. Chemicals and Reagents

Food pellets were purchased from Gold Coin, Malaysia. All blood collection tubes–EDTA and serum tubes (BD Vacutainer^®^), urine container, syringes and needle (25G, BD PrecisionGlide^™^) were from Becton, Dickinson and Company, Franklin Lakes, NJ, USA. Ketamine and xylazine were from Troy Laboratories PTY Limited, Glendenning, NSW, Australia. Formaldehyde solution, 37–38% and absolute ethanol were from HmbG^®^ Chemicals. Harris Haematoxylin and Eosin Y 1% solutions were from Labchem Sdn. Bhd., Petaling Jaya, Malaysia. Paraplast^®^Plus^™^ tissue embedding medium was from Leica Biosystems, Richmond, IL, USA. Digital picture exchange (D.P.X.) Mountant (R&M Marketing, Essex, UK) was purchased from Essen-Haus Sdn. Bhd., Selangor, Malaysia. ≥ 99.9% deuterated deuterium oxide (D_2_O), trimethylsilyl propionic acid-d4 sodium salt (TSP), non-deuterated potassium dihydrogen phosphate (KH_2_PO_4_), sodium deuterium oxide (NaOD), sodium azide (NaN_3_) were supplied by Merck (Darmstadt, Germany). 

### 3.2. Plant Extract Preparation

*C. nutans* was collected from the Sendayan Commodities Development Centre in Seremban, Negeri Sembilan, Malaysia. The sample was identified by Dr. Shamsul Khamis, an in-house botanist, and the voucher specimen (SK 2883/15) has been deposited in the herbarium of the Institute of Bioscience, Universiti Putra Malaysia. *C. nutans* leaves were subjected to air drying under the shade for 2 weeks until reaching a constant weight. The dried leaves were then milled into a fine powder with uniform particle size using a laboratory grinder (Waring, Stamford, CT, USA) and a 315 mm test sieve (Retsch, Haan, NW, Germany). The ground plant powder was then subjected to deionized water and ultrasound-assisted extraction. The plant sample to solvent ratio was kept constant i.e., 1 g of plant sample to 10 mL of solvent. Sonication was carried out for 1 h without heating using a HP heating series ultrasonic cleaners (Kudos, Shanghai, China). The ultrasonic frequency was set as 53 kHz. The extraction process was repeated thrice. Extracts were then filtered, vacuum evaporated, freeze dried and kept chilled at 4 °C until further analysis.

### 3.3. Experimental Animals

In total, 13 female Sprague Dawley rats were purchased from Saintik Enterprise, Malaysia. All rats were used at 9 weeks old, with weights from 180–250 g, and all experiments were approved by the Universiti Putra Malaysia Institutional Animal Care and Use Committee (AUP No. R080/2016). Animals were acclimatized for 1 week prior to the start of experiments. Rats were housed (3 animals per cage) in polycarbonate cages at 22–25 °C with food and water given ad libitum.

### 3.4. Acute Oral Toxicity Study

Acute toxicity testing was performed according to the OECD [43] for testing of chemicals. All experimental animals were randomly assigned into two groups—untreated and treated. Animals were fed a single dose of either distilled water (untreated) or water dissolved 5000 mg/kg of *C. nutans* water leaf extract at 10 mL/kg of body weight (bw) (treated) by oral gavage. The dose of 5000 mg/kg was chosen based on the highest recommended dose by the approaches of the OECD and Lorke [43,44]. Cage-side observation was conducted to evaluate for signs of toxicity at intervals of 30 min and 1, 2, 4, and 6 h as well as daily for 14 days. Physical observations, such as inactivity, isolation, not grooming, abnormal gait, persistent scratching, lethargy, not eating or drinking, eyes or nose discharge diarrhea and mortality, were considered signs of toxicity throughout the 14 days. On Day 15, all animals were sacrificed by intra-cardiac puncture under anesthesia using a ketamine/xylazine cocktail (91 mg/kg ketamine and 9.1 mg/kg xylazine) administered intraperitoneally at 10 mL/kg. Toe-pinch was conducted to assess the complete sedation of all animals before intra-cardiac puncture.

### 3.5. Body and Organ Weight Analysis

The body weights of all rats were recorded on Days 0 and 15, while the weights of all the major organs collected; heart, lungs, liver, spleen and kidneys were measured on Day 15 after the animals were sacrificed.

### 3.6. Food and Water Consumption

Food and water consumption were recorded on Days 1, 6 and 11 [45]. Briefly, rats were supplied with 250 mL of water and 100 g of food pellets the day before, and the remaining water and food pellets were measured the next day to determine the total consumption.

### 3.7. Hematology and Biochemical Analysis

Blood was collected from all animals by intra-cardiac puncture on Day 15 under anesthesia. Blood collected in ethylenediaminetetraacetic acid (EDTA) tubes was subjected to full blood profile analysis, while serum collected in plain vacutainers was subjected to biochemical analysis at the veterinary laboratory services unit, Faculty of Veterinary Medicine, Universiti Putra Malaysia. The biochemical parameters analyzed included total bilirubin, aspartate aminotransferase (AST), alanine aminotransferase (ALT) and alkaline phosphatase (ALP) for liver function testing and blood urea nitrogen and serum creatinine for renal function testing.

### 3.8. Histopathological Analysis

All animals were sacrificed, and major organs, such as the heart, lungs, liver, spleen and kidneys, were collected and fixed in 10% PBS-formalin for histopathological analysis. All organs were then grossly inspected and subjected to standard hemotoxylin and eosin (H & E) staining. Briefly, organs were grossly sliced and placed in their individual cassette holders and dehydrated gradually in 50% ethanol, 70% ethanol, 95% ethanol and 100% ethanol. Then the organs were oven dried overnight before being embedded in paraffin wax. Embedded organs were fixed and subjected to sectioning on a microtome. Organs were trimmed and then sectioned into 3 μm thick slices. The tissue sections were then mounted on glass slides and air dried overnight prior to staining. Slides were stained with hematoxylin dye, rinsed with deionized water followed by tap water to allow for stain development. The stained slides were dipped in acid ethanol to be destained and then rinsed a couple of times in tap water and deionized water before being counter stained with eosin. Slides were then dehydrated in xylene and affixed to slides using D.P.X. mountant covered with a coverslip for microscopy examination. All slides were examined using a light microscope (Leica DM2500, Wetzlar, Germany).

### 3.9. Serum and Urine Sample Preparation and ^1^H-NMR Spectra Acquisition and Processing

The biofluid preparation and the NMR acquisition were conducted according to procedures reported by Maulidiani et al. [23] with appropriate modification.

Serum samples were collected from the untreated and treated groups on the day before treatment (Day 0) and at the final time point of the experiment (Day 15) according to animal handling ethics and kept in plain red vacutainer tubes (without the addition of EDTA or anticoagulant) prior to ^1^H-NMR analysis. The collected whole blood was allowed to stand for 1 h at 4 °C. The blood was allowed to clot and centrifuged at 3100 rpm for 15 min at 4 °C. The resulting serum supernatant was then aliquoted into clean microcentrifuge tubes using a sterile pipette and stored at −80 °C prior to analysis. For serum ^1^H-NMR analysis, 200 μL of thawed serum was mixed with 400 μL of KH_2_PO_4_ buffer in D_2_O containing 0.2% of TSP. The pH was adjusted to pH 7.4 using 1 M NaOD. The ^1^H-NMR analysis was performed using a 500 MHz Varian INOVA NMR spectrometer (Varian Inc., California, CA, USA) at a frequency 499.90 MHz. Pre-saturation (PRESAT) was first performed to suppress the water peak signal. Carr–Purcell–Meiboom–Gill (CPMG) pulse sequence for T2 relaxation measurement was then applied to suppress the broad protein resonances, with 128 scans and a total acquisition time of 9 min 4 s.

Urine samples were collected from the untreated and treated groups’ rats, which were placed in individual metabolic cages the day before treatment (Day 0), 2 h after the treatment (Day 1), and on the post-treatment days (Days 2, 5, 10, and 15). Each cage urine tube was filled with 0.1% sodium azide as an anti-microbial agent. The collected urine samples were aliquoted into clean microcentrifuge tubes using a sterile pipette and stored at −80 °C prior to analysis. Prior to ^1^H-NMR analysis, urine samples were thawed and centrifuged at 4000 rpm for 10 min. A total of 400 μL of urine sample was then mixed with 200 μL of KH_2_PO_4_ buffer in D_2_O (pH 7.4) containing 0.1% of TSP. The ^1^H-NMR analysis was performed at a frequency of 499.90 MHz at room temperature (25 °C) using the PRESAT setting with 64 scans.

The acquired ^1^H-NMR spectra were then subjected to manual phasing, baseline correction, and a binning process using Chenomx software (Version 8.1, Alberta, AB, Canada). The signal at δ 4.76–4.97 (water) in serum samples was excluded. For urine samples, the excluded regions were δ 4.70–5.04 (water) and δ 5.55–5.95 (urea). Spectral data in the region of δ 0.50 to δ 10.00 were then normalized using the total spectral area normalization method and binned into a bin spectral width of 0.04 ppm, resulting in a total of 233 (serum) and 221 (urine) integrated regions per NMR spectrum. Spectral intensities were then scaled to TSP. The processed data were then subjected to SIMCA-P+ (Version 13.0, Umetrics AB, Umeå, Sweden) prior to MVA, and both unsupervised and supervised approaches were applied. Pareto scaling was applied to all data. Statistical differences in metabolites based on quantitative NMR spectra were calculated using Tukey’s test. 

### 3.10. Metabolic Pathway Analysis

A metabolic pathway map was established according to all the identified urinary metabolites, and the correlations were computed by referring to the Kyoto Encyclopaedia of Genes and Genomes (KEGG), a web-based free database resource [34].

## 4. Conclusions

In conclusion, single administration of 5000 mg/kg of *Clinacanthus nutans* extract did not lead to any abnormal acute toxicity signs during the 14 days of the observation period, based on both conventional (physical observation and hematological, biochemical and histopathological analyses) and advanced ^1^H-NMR serum and urinary metabolomics evaluations. Therefore, the LD_50_ of *C. nutans* water leaf extract is likely to be more than 5000 mg/kg bw rat.

## Figures and Tables

**Table 1:** Hematology analysis on the acute oral toxicity effect of *Clinacanthus nutans* water leaf extract in Sprague Dawley rats.

Parameter	Unit	Treatment	
		Untreated (Distilled Water)	Treated (*C. nutans* Water Extract 5000 mg/kg)
Red blood cells	10^12^/L	7.132 ± 0.367	6.811 ± 0.237
Hemaglobin	g/L	136.6 ± 5.307	131.143 ± 3.362
Packed cells volume	L/L	0.418 ± 0.013	0.381 ± 0.01 *, *p* = 0.0489
Mean corpuscular volume	fL	59.0 ± 1.581	56.142 ± 0.738
Mean corpuscular hemaglobin concentration	g/L	326.6 ± 4.925	344.0 ± 2.093 **, *p* = 0.0046
White blood cells	10^9^/L	9.650 ± 1.049	10.024 ± 1.345
B Neutrophils	10^9^/L	0.134 ± 0.021	0.146 ± 0.030
S Neutrophils	10^9^/L	1.912 ± 0.225	2.059 ± 0.387
Lymphocytes	10^9^/L	6.910 ± 0.861	7.047 ± 0.942
Monocytes	10^9^/L	0.536 ± 0.052	0.614 ± 0.116
Eosin	10^9^/L	0.166 ± 0.067	0.163 ± 0.041
Basophil	10^9^/L	0.000	0.000
Thrombocytes	10^9^/L	927.4 ± 29.763	837.429 ± 59.503
Plasma protein	g/L	70.0 ± 2.025	72.857 ± 1.639
Icterus index	U	2.0 ± 0.00	2.0 ± 0.00

Values are expressed as mean ± SEM (untreated, *n* = 6; treated, *n* = 7) where * *p* < 0.05 and ** *p* < 0.005 were considered significant.

**Table 2:** Biochemical analysis on the acute oral toxicity effect of *Clinacanthus nutans* water leaf extract in Sprague Dawley rats.

Parameter	Unit	Treatment	
		Untreated	Treated
**Liver Function Test**			
ALT	U/L	100.6 ± 36.57	94.17 ± 21.47
ALP	U/L	126.8 ± 11.46	116.9 ± 6.808
AST	U/L	340.3 ± 107.5	299.9 ± 42.69
Total Bilirubin	μmol/L	1.100 ± 0.1033	1.043 ± 0.1716
**Kidney Function Test**			
Creatinine	μmol/L	73.50 ± 4.478	70.86 ± 1.056
Urea	mmol/L	8.733 ± 0.4937	7.614 ± 0.9647

Values are expressed as mean ± SEM (untreated, *n* = 6; treated, *n* = 7).

**Table 3:** Comparison of the significant urinary metabolite level changes of treated rat on Day 0, 1 and 2 and their respective variable importance in the projection (VIP) and *p*-values.

Metabolites	δH ppm	VIP	Day 0	Day 1	Day 2	Day 1 vs. Day 0		Day 2 vs. Day 0		Day 2 vs. Day 1	
						Fold Change	*p*	Fold Change	*p*	Fold Change	*p*
Betaine	3.26	3.55	0.0188 ± 0.0038	0.0527 ± 0.0218	0.0243 ± 0.0076	2.803	0.002	1.290	0.115	0.460	0.007
Dimethylamine	2.70	2.72	0.0124 ± 0.0052	0.0253 ± 0.0124	0.0248 ± 0.0059	2.047	0.025	2.004	0.001	0.979	0.919
Cis aconitate	3.10	2.52	0.0071 ± 0.0017	0.0207 ± 0.0018	0.0138 ± 0.0060	2.899	0.000	1.932	0.016	0.667	0.013
Citrate	2.54	2.46	0.0206 ± 0.0096	0.0399 ± 0.0174	0.0306 ± 0.0009	1.933	0.025	1.484	0.072	0.768	0.238
1,2-propanediol	1.14	1.60	0.0026 ± 0.0004	0.0080 ± 0.0012	0.0029 ± 0.0010	3.106	0.000	1.143	0.389	0.368	0.000
Methylguanidine	2.82	1.27	0.0022 ± 0.0003	0.0059 ± 0.0012	0.0029 ± 0.0008	2.726	0.000	1.346	0.032	0.494	0.000
Methylamine	2.58	1.16	0.0020 ± 0.0004	0.0060 ± 0.0040	0.0046 ± 0.0028	2.942	0.023	2.263	0.031	0.769	0.467
Lactate	1.30	0.94	0.0036 ± 0.0007	0.0061 ± 0.0016	0.0038 ± 0.0011	1.679	0.003	1.044	0.752	0.622	0.009
*N*,*N*-dimethylglycine	2.90	0.72	0.0032 ± 0.0005	0.0051 ± 0.0012	0.0036 ± 0.0019	1.604	0.002	1.124	0.610	0.701	0.098
Acetoacetate	2.30	0.65	0.0040 ± 0.0015	0.0060 ± 0.0007	0.0046 ± 0.0027	1.499	0.008	1.164	0.584	0.777	0.230
Unknown U1	3.30	2.48	0.0024 ± 0.0003	0.0158 ± 0.0029	0.0056 ± 0.0016	6.580	0.000	2.307	0.000	0.351	0.000
Unknown U2	2.74	1.03	0.0028 ± 0.0010	0.0046 ± 0.0008	0.0022 ± 0.0004	1.680	0.002	0.801	0.199	0.477	0.000

Values are the means ± standard deviation based on seven replicates. Values of fold change > 1 represent increase and < 1 represent decrease. Value of *p* < 0.001 is very significant, 0.05 ≤ *p ≤* 0.001 shows significant and *p* > 0.05 is not significant.

## Supplementary Materials

The following are available online, Table S1: Metabolites identified from ^1^H-NMR serum and urinary spectra.
