# Supplementary material for: Hematological, Biochemical, Histopathological and 1H-NMR Metabolomics Application in Acute Toxicity Evaluation of Clinacanthus nutans Water Leaf Extract
Source: Molecules. 2018 Aug 29;23(9):2172. doi: 10.3390/molecules23092172 (PMC6225189; doi:10.3390/molecules23092172)
Supplement: Supplementary file 1 [file molecules-23-02172-s001.pdf]

Hematological, biochemical, histopathological and ^{1}H-NMR metabolomics application in acute toxicity evaluation of *Clinacanthus nutans* water leaf extract

Leng Wei Khoo[1], Audrey Siew Foong Kow[2], M. Maulidiani[3], Ming Tatt Lee[4], Chin Ping Tan[5], Khozirah Shaari[3], Chau Ling Tham[2], Faridah Abas[1,3*]

[1] Department of Food Science, Faculty of Food Science and Technology, Universiti Putra Malaysia, 43400 Serdang, Selangor, Malaysia.

[2] Department Biomedical Science, Faculty of Medicine and Health Sciences, Universiti Putra Malaysia, 43400 Serdang, Selangor, Malaysia

[3] Laboratory of Natural Products, Institute of Bioscience, Universiti Putra Malaysia, 43400 Serdang, Selangor, Malaysia.

[4] Faculty of Pharmaceutical Sciences, UCSI University Kuala Lumpur Campus, Jalan Menara Gading, UCSI Heights (Taman Connaught), Cheras 56000 Kuala Lumpur, Malaysia

[5] Department of Food Technology, Faculty of Food Science and Technology, Universiti Putra Malaysia, 43400 Serdang, Selangor, Malaysia.

*Corresponding author:
Faridah Abas, PhD, e-mail: faridah_abas@upm.edu.my, tel: +603 89468343

Supplementary materials

Table S1. Metabolites identified from ^{1}H-NMR serum and urinary spectra.

No	Metabolites	Chemical shifts	Biofluids	
			Serum	Urine
1	Formate	8.46 (s)	+	+
2	Histidine	7.82 (s), 7.07 (s), 3.98 (m), 3.24-3.21 (m), 3.14-3.09 (m)	+	-
3	Phenylalanine	7.42 (m), 7.32 (d, *J* =7.5 Hz), 4.00 (m), 3.28 (m), 3.14 (m)	+	-
4	Tyrosine	7.18 (m), 6.90 (d, *J* = 8.0 Hz)	+	-
5	Glucose	5.24 (d, *J* = 3.5 Hz), 4.67 (d, *J* = 8.0 Hz), 3.92 (dd, *J* =12.0, 2.0 Hz), 3.86-3.89 (m), 3.78-3.70 (m), 3.55-3.52 (m), 3.51-3.45 (m), 3.43-3.38 (m), 3.24 (dd, *J* = 9.5, 8.0 Hz)	+	-
6	Lactate	4.10 (q, *J* =7.0 Hz), 1.32 (d, *J* =6.5 Hz)	+	+
7	Glycerol	3.78 (m), 3.65 (dd, *J* =12.0, 4.5 Hz), 3.58 (m)	+	-
8	Creatine	3.94 (s)	+	+
9	Choline	3.20 (s)	+	-
10	Dimethyl sulfone	3.14 (s)	+	+
11	*N,N*-dimethylglycine	3.73 (s), 2.93 (s)	+	+
12	Citrate	2.68 (d, *J* = 16.0 Hz), 2.54 (d, *J* = 16.0 Hz)	+	+
13	Glutamine	2.14 (m), 2.45 (m)	+	-
14	Succinate	2.41 (s)	+	+
15	Pyruvate	2.37 (s)	+	-
16	3-hydroxybutyrate	1.22 (d, *J* = 6.5 Hz), 2.31 (m), 2.42 (m)	+	-
17	Acetoacetate	3.45 (s), 2.30 (s)	+	+
18	Acetone	2.23 (s)	+	-
19	*N*-acetylornithine	2.04 (s)	+	-
20	Acetate	1.92 (s)	+	+
21	Alanine	1.46 (d, *J* = 7.0 Hz), 3.82 (q, *J* = 7.0 Hz)	+	+
22	3-hydroxyisobutyrate	1.07 (d, *J* = 7.0 Hz)	+	-
23	Valine	1.04 (d, *J* = 7.5 Hz), 0.99 (d, *J* = 7.5 Hz)	+	-
24	Isoleucine	1.01 (d, *J* = 7.0 Hz), 0.94 (t, *J* = 7.0 Hz)	+	-
25	Leucine	0.95 (m), 1.70 (m)	+	-
26	Dimethylamine	2.73 (s)	+	+
27	Unknown S1	1.18 (t, *J* = 7.0 Hz)	+	-
28	Trigonelline	9.13 (s), 8.84 (m), 8.09 (t, *J* = 8.0 Hz), 4.44 (s)	-	+
29	1-methylnicotinamide	9.28 (s), 8.97 (d, *J* = 5.5 Hz), 8.90 (d, *J* = 6.5 Hz), 8.19 (t, *J* = 8.5 Hz),	-	+

		4.46 (s)		
30	Allantoin	6.04 (s), 5.38 (s)	-	+
31	Methylguanidine	2.82 (s)	-	+
32	Methylamine	2.60 (s)	-	+
33	Pyridoxine	7.66 (s)	-	+
34	Cis-aconitate	3.10 (s)	-	+
35	2-oxoglutarate	3.01 (t, *J* = 6.5 Hz), 2.44 (t, *J* = 6.5 Hz)	-	+
36	Fumarate	6.52 (s)	-	+
37	2-aminobutyrate	1.87 (m), 0.97 (t, *J* =7.5 Hz)	-	+
38	Glycine	3.57 (s)	-	+
39	Urea	5.77 (br. s)	-	+
40	Creatinine	4.02 (s), 3.05 (s)	-	+
41	Betaine	3.91 (s), 3.27 (s)	-	+
42	Phosphocholine	3.21 (s)	-	+
43	Taurine	3.43 (t, *J* = 6.5 Hz), 3.27 (t, *J* = 6.5 Hz)	-	+
44	4-hydroxyphenylacetate	7.17 (d, *J* = 8.5 Hz), 6.85 (d, *J* = 8.5 Hz), 3.44 (s)	-	+
45	Hippurate	8.52 (s), 7.82 (d, *J* = 7.5 Hz), 7.64 (t, *J* = 7.5 Hz), 7.55 (t, *J* = 8.0 Hz), 3.98 (d, *J* = 5.5 Hz)	-	+
46	Benzoate	7.88 (d, *J* = 7.0 Hz), 7.49 (t, *J* = 7.5 Hz), 7.55 (t, *J* =8.0 Hz)	-	+
47	*N*-phenylacetylglycine	8.02 (s), 7.42 (t, *J* = 8.0 Hz), 7.37 (m), 3.75 (d, *J* = 5.5 Hz), 3.68 (s)	-	+
48	N6-acetyl-lysine	1.96 (s), 1.54 (m), 1.42 (s)	-	+
49	1,2-propanediol	1.14 (d, *J* = 6.5 Hz)	-	+
50	3-indoxylsulfate	7.71 (d, *J* = 8.0 Hz), 7.51 (d, *J* = 8.5 Hz), 7.35 (s), 7.26 (t), 7.22 (m)	-	+
51	Unknown U1	3.30 (s)	-	+
52	Unknown U2	2.74 (s)	-	+

+: Detected; -: No detected
